# An Observational Study of Cardiovascular Outcomes of Tirzepatide vs Glucagon-Like Peptide-1 Receptor Agonists

**DOI:** 10.1016/j.jacadv.2025.101740

**Published:** 2025-05-28

**Authors:** Sourbha S. Dani, Bhargav Makwana, Sumanth Khadke, Ashish Kumar, Pardeep Jhund, Khurram Nasir, Naveed Sattar, Sadeer Al-Kindi, Gregg Fonarow, Javed Butler, Deepak L. Bhatt, Mikhail N. Kosiborod, Anju Nohria, Sarju Ganatra

**Affiliations:** a Division of Cardiovascular Medicine, Department of Medicine, Lahey Hospital and Medical Center, Beth Israel Lahey Health, Burlington, Massachusetts, USA; b Department of Medicine, Cleveland Clinic, Akron, Ohio, USA; c BHF Cardiovascular Research Centre, School of Cardiovascular and Metabolic Health, University of Glasgow, Glasgow, Scotland, United Kingdom; d Division of Cardiovascular Prevention and Wellness, Houston Methodist DeBakey Heart and Vascular Center, Houston, Texas, USA; e Division of Cardiology, Department of Medicine, Ronald Reagan-UCLA Medical Center, Los Angeles, California, USA; f Baylor Scott and White Research Institute, Dallas, Texas, USA; g Mount Sinai Fuster Heart Hospital, Icahn School of Medicine at Mount Sinai, New York, New York, USA; h Saint Luke's Mid America Heart Institute, University of Missouri, Kansas City, Missouri, USA; i Brigham and Women's Hospital Heart and Vascular Center, Harvard Medical School, Boston, Massachusetts, USA

**Keywords:** cardiovascular outcomes, GLP-1 receptor agonists, tirzepatide

## Abstract

**Background:**

While cardiovascular benefits of tirzepatide, a glucose-dependent insulinotropic peptide/glucagon-like peptide-1 receptor agonist in patients with type 2 diabetes mellitus (T2DM), and its comparative effectiveness vs glucagon-like peptide-1 receptor agonists (GLP-1RAs) is studied in randomized controlled trials, real-world outcomes may provide critical insights.

**Objectives:**

The purpose of this study was to examine the cardiovascular benefits of tirzepatide vs GLP-1RA in people living with overweight or obesity, with T2DM, age ≥40 years, and pre-existing ischemic heart disease (IHD).

**Methods:**

A retrospective cohort analysis of de-identified, aggregate patient data from the TriNetX research network was conducted. People with T2DM, age ≥40 years, pre-existing IHD, and body mass index ≥25 kg/m^2^ receiving either tirzepatide or GLP-1RA were identified and divided into 2 groups (tirzepatide vs GLP-1RA). After propensity score matching, Cox-proportional HRs were used to compare efficacy and safety outcomes during 1-year follow-up.

**Results:**

Among 47,719 adults, 753 received tirzepatide, and 46,966 were on GLP-1RA. After propensity score matching, each group had 751 adults (mean age 59.9 ± 8.9 years, 46.5% females, 74.8% White adults in the tirzepatide group). Treatment with tirzepatide was associated with lower primary composite outcomes of acute myocardial infarction, ischemic stroke, and all-cause mortality (HR: 0.60, 95% CI: 0.43-0.84, *P* < 0.001). Individually, acute myocardial infarction (HR: 0.59, 95% CI: 0.38-0.91) and all-cause mortality (HR: 0.35, 95% CI: 0.14-0.88, *P* = 0.001) were also found to be favorable in the tirzepatide group.

**Conclusions:**

Tirzepatide use is associated with better outcomes in adults aged 40 years or older with T2DM, body mass index ≥25 kg/m^2^, and pre-existing IHD.

Despite preventive, pharmacotherapeutic, and invasive management advances, cardiometabolic diseases dominate with significant morbidity, mortality, and substantial economic loss globally.^1^ In the United States, coronary heart disease was responsible for 41.2% of cardiovascular disease (CVD) attributable deaths in 2020, accounting for 12% of total health expenditures.^2^ The burden of CVD is exacerbated by the rising incidence of type 2 diabetes mellitus (T2DM) and obesity, both significant risk factors for CVD. In 2021, 529 million individuals were estimated to have T2DM globally; obesity was attributed to 52.2% of T2DM-associated Disability Adjusted Life Years, highlighting unmet critical needs to target T2DM and obesity.^3^

The last decade has seen tremendous pharmacotherapeutic progress in addressing T2DM and obesity. A plethora of new antidiabetic medications, particularly glucagon-like peptide-1 receptor agonists (GLP-1RAs), have demonstrated positive cardiometabolic effects with improved glycemic control, weight loss, blood pressure control, and lower inflammation and thus have proven benefits in reducing rates of nonfatal acute myocardial infarction (AMI), stroke, and cardiovascular death.^4^ Studies have shown that GLP-1RA significantly reduce major adverse cardiovascular events (MACEs) in patients living with or without T2DM, regardless of pre-existing CVD, and are effective in reducing cardiovascular and all-cause mortality.^5^, ^6^, ^7^, ^8^ As a result, GLP-1RA are one of the 2 preferred glucose-lowering agents in patients with T2DM and CVD or at increased risk of CVD by various societal guidelines.^9^^,^^10^ Since the first approval of exenatide in 2005, the GLP-1RA pipeline has grown and has demonstrated superiority of newer GLP-1RA, such as liraglutide and semaglutide, related to robust GLP-1R target engagement. Several strategies were attempted to enhance the efficacy of GLP-1RA, including dose uptitration. However, these efforts were limited due to gastrointestinal (GI) side effects and only moderate improvements in weight loss and glycemic control.

The introduction of tirzepatide, a combined glucose-dependent insulinotropic polypeptide (GIP) and GIP/GLP-1RA, has further strengthened the cardiometabolic field. Tirzepatide is a novel, unimolecular twincretin agonist of GIP-GLP-1RA, which, after initial phase 2 studies,^11^^,^^12^ was found to be superior in glycaemia reduction compared to the standard of care or placebo in the SURPASS RCT program.^13^ Tirzepatide not only improved glycemia and body weight but also slowed the reduction in estimated glomerular filtration rate (eGFR)^14^ and is beneficial in metabolic dysfunction–associated fatty liver disease.^15^^,^^16^ When compared with semaglutide 1 mg directly^17^ and indirectly,^18^ glycemic control, weight reduction, blood pressure control, and lipid reduction were better with tirzepatide. In a prespecified meta-analysis of the SURPASS trials,^19^ tirzepatide was found to be safe from a cardiovascular standpoint with a trend towards lower MACE. However, the published SURPASS trials were not powered to detect statistically significant differences in MACE due to low event rates.

To date, there are no head-to-head RCTs comparing the effect of tirzepatide vs contemporary GLP-1RA on cardiovascular outcomes. While this question is being addressed by the SURPASS-CVOT (A Study of Tirzepatide Compared With Dulaglutide on Major Cardiovascular Events in Participants With Type 2 Diabetes)^20^ trial that should report in 2025, this knowledge gap can be estimated by real-world evidence-based observational studies.

In this observational cohort study, we aim to explore the cardiovascular outcomes of tirzepatide vs contemporary GLP-1RAs using a large research network database.

## Methods

### Data source and patient population

A retrospective observational cohort analysis was conducted using TriNetX Global Research Network data queried from January 1, 2022 until December 31, 2022. The TriNetX Global Research Network offers access to inpatient and outpatient electronic health records (EHRs) of approximately 110 million individuals, derived mainly from US healthcare institutions. This platform only has aggregate, de-identified data per the de-identification standard defined in Section §164.514(a) of the HIPAA Privacy Rule. This research utilized anonymized patient data and was thus exempted by the Institutional Review Board of Lahey Hospital and Medical Center.

People living with with T2DM, age ≥40 years, IHD, BMI ≥25 kg/m^2^, and receiving either tirzepatide or a GLP-1RA (semaglutide, liraglutide, dulaglutide, lixisenatide) were identified using International Classification of Disease-10th revision (ICD-10) codes and EHR curated data. The population was further stratified into 2 groups based on tirzepatide and GLP-1RA use. The index date for the study's follow-up was the date of commencement of tirzepatide or GLP-1RA treatment for each group. The retrieval window for the baseline characteristics of study participants was set to 20 years before the index event date. The Current Procedural Terminology and ICD-10 codes used to identify the cohorts and study window definitions are available in the Supplemental Tables 1 to 5. Data analysis was performed on January 3, 2024. This study was reported per the Strengthening the Reporting of Observational Studies in Epidemiology (STROBE) guidelines.

### Study outcomes

The primary outcome assessed in this study was a composite of AMI, stroke, and all-cause mortality. These outcomes were selected to explicitly emulate the ongoing SURPASS-CVOT for eligibility and outcomes criteria to overcome the possible distortion of results by baseline confounders.^21^ Secondary outcomes included the individual components of the primary composite outcome. Secondary outcomes also included all-cause hospitalization or ER visits, heart failure exacerbations (HFEs), new-onset atrial fibrillation or flutter, pulmonary hypertension, acute kidney injury (AKI), and the need for new-onset renal replacement therapy. Laboratory parameters such as C-reactive protein levels ≥5 mg/dL, low-density lipoprotein (LDL) ≤70 mg/dL, triglyceride ≤150 mg/dL, albumin:creatinine ratio ≤30 mg/g, and albumin:creatinine ratio ≤300 mg/g were extracted from the database. All outcomes were assessed during a 12-month follow-up period. HFE was defined using ICD-10 codes as either a requirement for intravenous diuretics or a diagnosis of pulmonary edema. Multiple safety outcomes/adverse events were assessed for both groups, including GI symptoms, gallbladder and pancreatic disorders, and nonalcoholic fatty liver disease (NAFLD)/hepatic fibrosis. In addition, the occurrence of influenza, pneumonia, and diabetic retinopathy were compared between the 2 groups. A subgroup analysis was conducted between tirzepatide vs semaglutide or liraglutide for all primary and secondary outcomes.

### Statistical analysis

Continuous variables are presented as mean ± SD or median (IQR), and categorical variables are presented as number (%). Baseline characteristics in the 2 groups were compared using independent-sample *t*-tests for continuous variables and chi-square tests for categorical variables. 1:1 propensity score matching (PSM) using a number of baseline demographic variables, comorbidities, medications, laboratory parameters, and prior healthcare utilization characteristics, as listed in Table 1 and Supplemental Table 5, was performed using greedy nearest-neighbor matching with a caliper of 0.1 times the pooled standard deviation of the linear propensity scores to control for baseline differences between the study groups. The standard mean difference is a quantitative method used to represent the difference between the mean of 2 groups in terms of standard deviation units to assess the balance in measured variables in the sample weighted by the inverse probability of treatment. The variables were chosen because of their potential impact on overall and cardiovascular outcomes.

**Table 1:** Baseline Characteristics of Population Before and After Propensity Score Matching

	Before PSM			After PSM		
	Tirzepatide (n = 753)	GLP-1RA (n = 46,966)	Std. Diff.	Tirzepatide (n = 751)	GLP-1RA (n = 751)	Std. Diff.
Demographics						
Age, y	59.9 ± 8.9	63.3 ± 10.2	0.352	59.9 ± 8.9	60.1 ± 9.6	0.019
Female	350 (46.5)	20,741 (44.2)	0.047	349 (46.5)	340 (45.3)	0.024
Non-Hispanic	675 (89.6)	37,953 (80.8)	0.251	673 (89.6)	684 (91.1)	0.050
White	564 (74.9)	31,781 (67.7)	0.160	562 (74.8)	577 (76.8)	0.047
BMI	37.6 ± 6.5	35.0 ± 6.6	0.398	37.6 ± 6.5	36.6 ± 6.3	0.100
Comorbidities						
Hypertension	712 (94.6)	44,006 (93.7)	0.036	710 (94.5)	705 (93.9)	0.029
Hyperlipidemia	718 (95.4)	42,037 (89.5)	0.222	716 (95.3)	711 (94.7)	0.031
Acute myocardial infarction	144 (19.1)	9,538 (20.3)	0.030	143 (19.0)	148 (19.7)	0.017
Ischemic stroke	54 (7.2)	4,454 (9.5)	0.084	54 (7.2)	42 (5.6)	0.065
History of prior PCI	101 (13.4)	4,757 (10.1)	0.102	101 (13.4)	99 (13.2)	0.008
Atrial fibrillation/flutter	127 (16.9)	8,728 (18.6)	0.045	127 (16.9)	131 (17.4)	0.014
Heart failure	219 (29.1)	14,387 (30.6)	0.033	219 (30.6)	204 (27.2)	0.044
Chronic kidney disease	199 (26.4)	14,895 (31.7)	0.117	198 (26.4)	188 (25.0)	0.030
Peripheral arterial disease	236 (31.3)	14,749 (31.4)	0.001	234 (31.2)	233 (31.0)	0.003
Chronic lower respiratory diseases	308 (40.9)	17,236 (36.7)	0.086	307 (40.9)	311 (41.4)	0.011
Malignancy	97 (12.9)	4,688 (10.0)	0.091	97 (12.9)	79 (10.5)	0.075
Medications						
Statin	707 (93.9)	42,547 (90.6)	0.124	705 (93.9)	698 (92.9)	0.038
ACE inhibitors	451 (59.9)	28,627 (61.0)	0.022	450 (59.9)	438 (58.3)	0.033
ARB	373 (49.5)	19,667 (41.9)	0.154	371 (49.4)	366 (48.7)	0.013
ARNi	48 (6.4)	1,468 (3.1)	0.153	46 (6.1)	52 (6.9)	0.032
Beta-blockers	619 (82.2)	36,988 (78.8)	0.087	617 (82.2)	610 (81.2)	0.024
Antiarrhythmics	598 (79.4)	31,055 (66.1)	0.302	596 (79.4)	593 (79.0)	0.010
Loop diuretics	342 (45.4)	21,308 (45.4)	0.001	340 (45.3)	330 (43.9)	0.027
Thiazide diuretics	366 (48.6)	21,361 (45.5)	0.063	364 (48.5)	361 (48.1)	0.008
Potassium-sparing diuretics	205 (27.2)	9,336 (19.9)	0.174	203 (27.0)	211 (28.1)	0.024
Empagliflozin	276 (36.7)	6,910 (14.7)	0.519	274 (36.5)	262 (34.9)	0.033
Dapagliflozin	128 (17.0)	3,041 (6.5)	0.331	126 (16.8)	124 (16.5)	0.007
Canagliflozin	75 (10.0)	3,146 (6.7)	0.118	75 (10.0)	56 (7.5)	0.090
Insulin	547 (72.6)	34,403 (73.3)	0.014	545 (72.6)	534 (71.1)	0.033
Metformin	605 (80.3)	34,845 (74.2)	0.147	603 (80.3)	621 (82.7)	0.062
Glipizide	176 (23.4)	12,241 (26.1)	0.062	176 (23.4)	191 (25.4)	0.046
Aspirin	604 (80.2)	35,964 (76.6)	0.088	602 (80.2)	602 (80.2)	<0.001
Clopidogrel	194 (25.8)	14,203 (30.2)	0.100	193 (25.7)	205 (27.3)	0.036
Ticagrelor	86 (11.4)	3,144 (6.7)	0.165	85 (11.3)	79 (10.5)	0.026
Warfarin	66 (8.8)	5,644 (12.0)	0.107	65 (8.7)	67 (8.9)	0.009
Apixaban	104 (13.8)	4,394 (9.4)	0.140	103 (13.7)	107 (14.2)	0.015
Rivaroxaban	70 (9.3)	2,680 (5.7)	0.137	69 (9.2)	69 (9.2)	<0.001
Linagliptin	52 (6.9)	2,782 (5.9)	0.040	52 (6.9)	33 (4.4)	0.11
Saxagliptin	15 (2.0)	991 (2.1)	0.008	15 (2.0)	24 (3.2)	0.075
Alogliptin	10 (1.3)	439 (0.9)	0.037	10 (1.3)	10 (1.3)	<0.001
Sitagliptin	163 (21.6)	10,374 (22.1)	0.011	163 (21.7)	163 (21.7)	<0.001
Lab values						
Creatinine (mg/dL)	1.0 ± 0.6	1.3 ± 4.2	0.094	1.0 ± 0.6	1.1 ± 0.6	0.036
LVEF <45%	44 (5.8)	1,579 (3.4)	0.119	43 (5.7)	45 (6.0)	0.011
BNP >150 pg/mL	80 (10.6)	5,337 (11.4)	0.024	80 (10.7)	80 (10.7)	<0.001
NT-proBNP >450 pg/mL	49 (6.5)	2,970 (6.3)	0.007	48 (6.4)	53 (7.1)	0.027
LDL cholesterol >130 mg/dL	257 (34.1)	12,280 (26.1)	0.175	256 (34.1)	267 (35.6)	0.031
Triglyceride	190.2 ± 144.1	189.3 ± 160.7	0.006	190.2 ± 144.3	189.6 ± 125.2	0.005
Hemoglobin A1c ≥7%	599 (79.5)	32,392 (69.0)	0.244	597 (79.5)	607 (80.8)	0.033
Aspartate aminotransferase	25.0 ± 13.1	25.6 ± 30.9	0.025	25.0 ± 13.1	25.8 ± 16.5	0.052
Alanine aminotransferase	28.1 ± 15.5	28.7 ± 29.1	0.026	28.1 ± 15.5	30.5 ± 23.6	0.120
CRP ≥5 mg/L	147 (19.5)	8,206 (17.5)	0.053	146 (19.4)	162 (21.6)	0.053
Prior healthcare utilization						
PCI	101 (13.4)	4,757 (10.1)	0.102	101 (13.4)	99 (13.2)	0.008
Outpatient visits	597 (79.3)	40,192 (85.6)	0.166	595 (79.2)	596 (79.4)	0.003
ER visits	427 (56.7)	23,855 (50.8)	0.119	425 (56.6)	433 (57.7)	0.022
Inpatient admissions	372 (49.4)	21,095 (44.9)	0.090	370 (49.3)	366 (48.7)	0.011

Values are mean ± SD or n (%).

ACE = angiotensin-converting enzyme; ARB = angiotensin receptor blocker; ARNi = angiotensin receptor neprilysin inhibitor; BNP = brain natriuretic peptide; CRP = C-reactive protein; ER = emergency room; GLP-1RA = glucagon-like peptide-1 receptor agonist; LDL = low-density lipoprotein; LVEF = left ventricular ejection fraction; NT-proBNP = N-terminal pro-B-type natriuretic peptide; PCI = percutaneous coronary intervention; PSM = propensity score matching.

After PSM, adjusted outcomes were compared between the 2 cohorts. Kaplan-Meier curves and Cox-proportional Hazard models were used for survival analysis. Statistical significance was set at a *P* value of <0.05. Statistical analyses were performed using integrated R for statistical computing on the TriNetX platform.

#### Sensitivity analyses

To increase the robustness of observational data, we performed a “look back” 12-month healthcare utilization of tirzepatide vs GLP-1RA groups for outpatient and emergency room visits and hospitalizations before December 31, 2022. We assessed falsification outcomes in the form of urinary tract infections, peptic ulcer disease, and ambulatory visits in the same follow-up time frame. Furthermore, we used the E-value measurement of E-value^22^ for primary and secondary outcomes to evaluate significant confounding and is noted in the tables. A higher E-value implies that a stronger unmeasured confounder would be required to explain away or nullify the observed association between the exposure and the outcome.

## Results

### Patient population

The study cohort included 47,719 adults. Among these, 753 were on tirzepatide, and 46,966 were on GLP-1RA. After PSM, 751 patients remained in each group and were included in the analysis (Supplemental Table 4).

The baseline characteristics of the study patients, before and after PSM, are shown in Table 1. The ICD/International Classification of Diseases, Anatomical Therapeutic Chemical Classification, or Veterans Affairs codes for these baseline characteristics are presented in Supplemental Table 5. Before PSM, patients receiving tirzepatide were younger than those receiving GLP-1RA, predominantly non-Hispanic and White adults. Patients receiving tirzepatide had a relatively higher prevalence of hypertension and hyperlipidemia. In contrast, a prior history of ischemic stroke, chronic kidney disease (stage 3 and above), and atrial fibrillation was noted more in the GLP-1RA group. The use of statin, angiotensin receptor blocker, beta-blockers, anti-arrhythmics, potassium-sparing diuretics, and sodium-glucose co-transporter-2 inhibitor (SGLT-2i) was noted more in patients receiving tirzepatide; however, after PSM, the 2 cohorts were well matched for demographics, comorbidities, medication use at baseline, laboratory values, and prior healthcare utilization.

### Outcomes

#### Primary outcome

**Figure 1:**
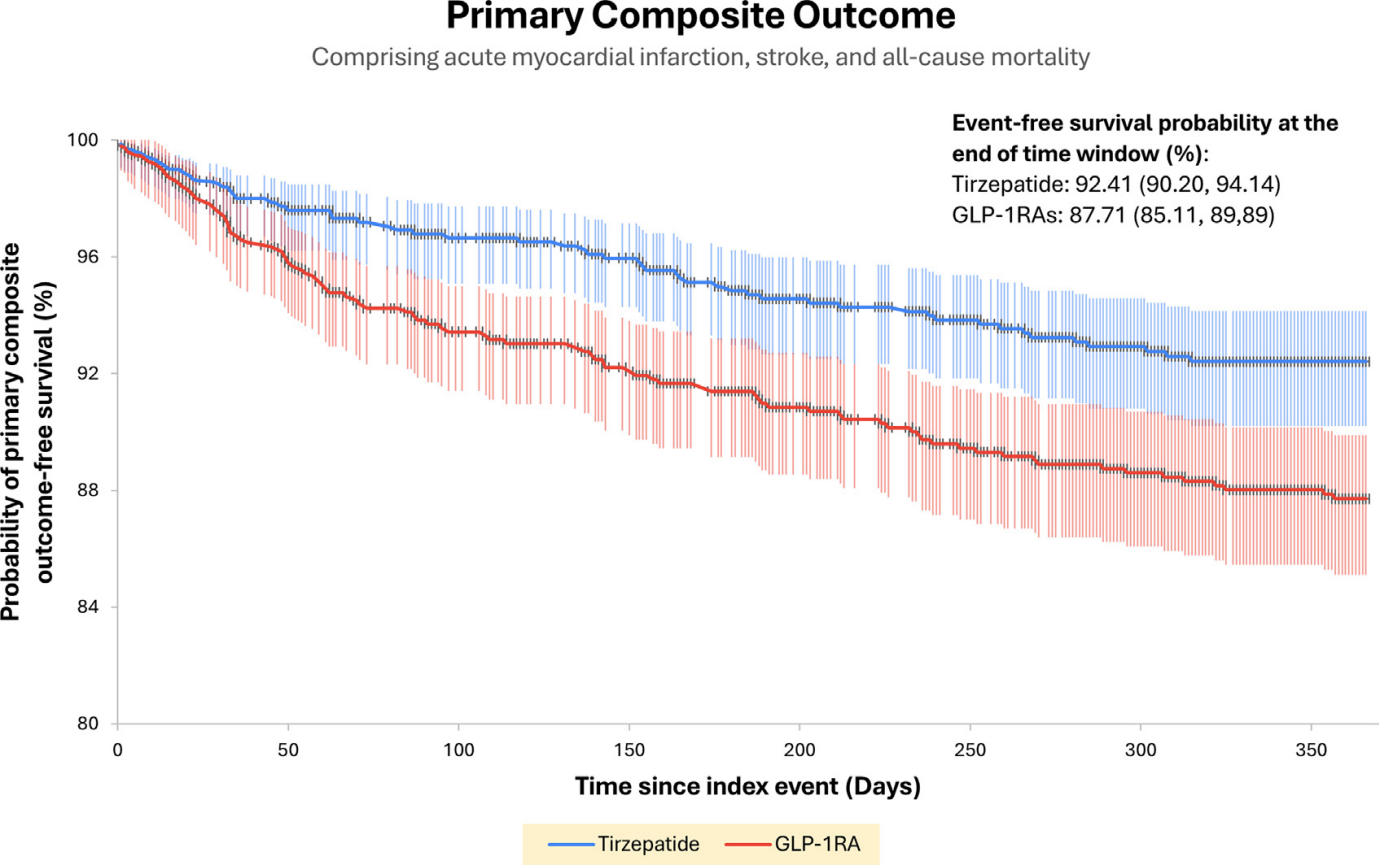
Kaplan-Meier Plot Showing Primary Composite Outcome-Free Survival in Patients on Tirzepatide vs GLP-1 Receptor Agonists

Tirzepatide was associated with a lower primary composite outcome of AMI, stroke, and all-cause mortality compared to GLP-1RA (relative risk reduction [RRR]: 40%, HR: 0.60 [95% CI: 0.42-0.84], *P* = 0.003) (Figure 1).

#### Secondary clinical outcomes

**Figure 2:**
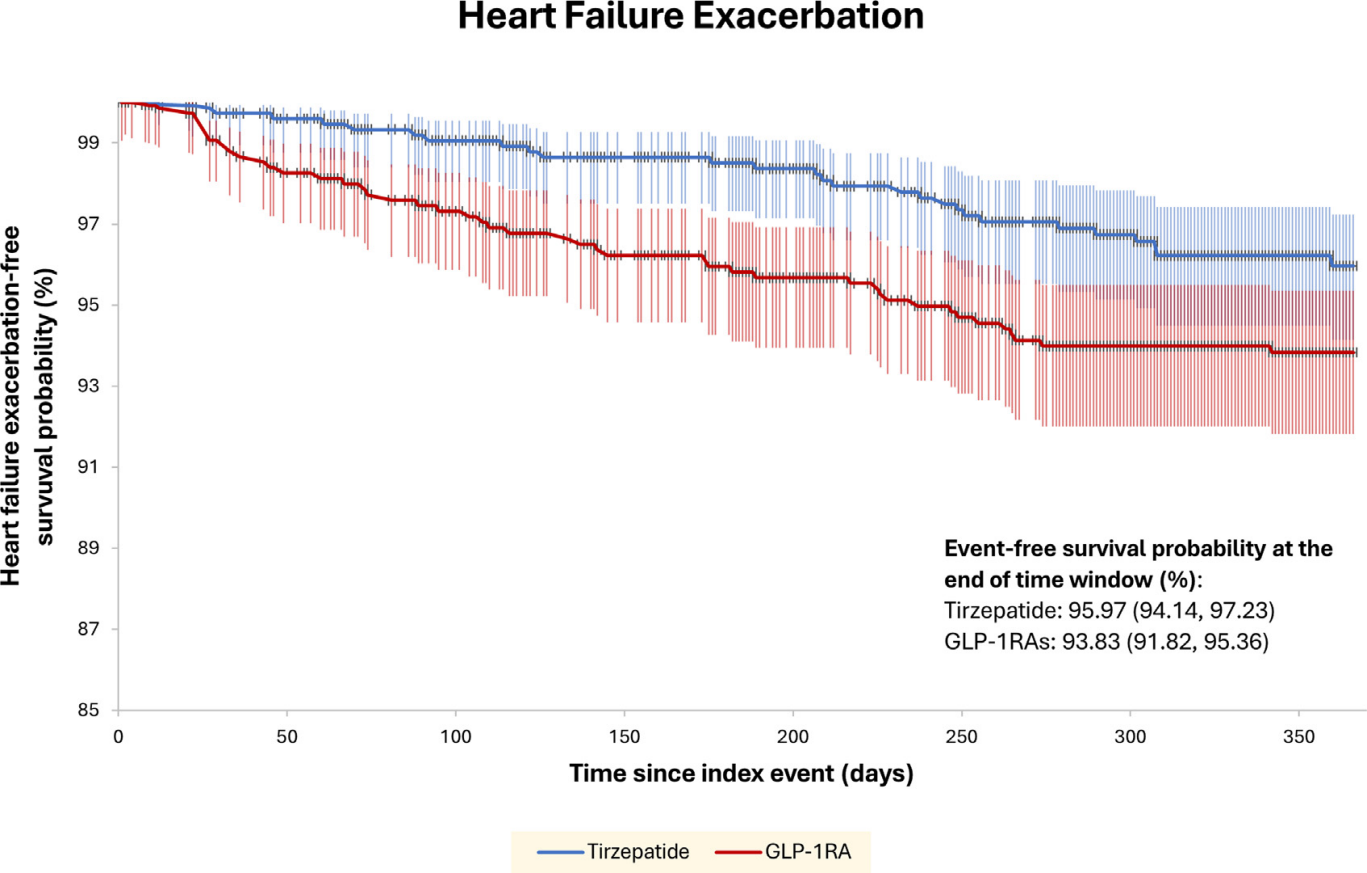
Kaplan-Meier Plot Showing Heart Failure Exacerbation-Free Survival in Patients on Tirzepatide vs GLP-1 Receptor Agonists

Among the individual components of the primary composite outcome, AMI (RRR: 42%, HR: 0.59 [95% CI: 0.38-0.91], *P* = 0.016) and all-cause mortality (RRR: 44%, HR: 0.35 [95% CI: 0.14-0.89], *P* = 0.021) were also significantly lower in adults receiving tirzepatide than in those receiving GLP-1RA. Although the incidence of ischemic stroke was less in the tirzepatide group (RRR: 19%, HR: 0.81 [95% CI: 0.45-1.43], *P* = 0.468), this finding did not achieve statistical significance. Other clinically relevant secondary endpoints which were significantly lower in the tirzepatide group included HFE (RRR: 40%, HR: 0.60 [95% CI: 0.37-0.98], *P* = 0.04) (Figure 2), new systolic heart failure (RRR: 26%, HR: 0.73 [95% CI: 0.54-0.99], *P* = 0.045), new-onset atrial fibrillation or atrial flutter (RRR: 45%, HR: 0.23 [95% CI: 0.07-0.68], *P* = 0.004), and new-onset AKI (RRR: 33%, HR: 0.67 [95% CI: 0.47-0.96], *P* = 0.028).

#### Laboratory outcomes

Tirzepatide was associated with a higher likelihood of achieving HbA1c ≤7 (HR: 1.50 [95% CI: 1.30-1.74], *P* < 0.001), LDL ≤70 mg/dL (HR: 1.32 [95% CI: 1.11-1.57], *P* = 0.002), triglyceride ≤150 mg/dL (HR: 1.26 [95% CI: 1.07-1.49], *P* = 0.006), albumin:creatinine ratio ≤30 mg/g (HR: 1.75 [95% CI: 1.11-2.77], *P* = 0.014), and albumin:creatinine ratio ≤300 mg/g (HR: 1.56 [95% CI: 1.08-2.25], *P* = 0.017) than GLP-1RA. Reduction in C-reactive protein was also less associated with the tirzepatide group than with the GLP-1RA group; however, this was not statistically significant (HR: 0.76 [95% CI: 0.38-1.52], *P* = 0.44). Weight loss, measured by the incidence of BMI ≤30, was comparable in both groups (HR: 0.93 [95% CI: 0.760-1.14], *P* = 0.492) (Table 2).

**Table 2:** Comparison of Efficacy Outcomes Among Patients on Tirzepatide vs GLP-1RA

Outcomes^a^	Tirzepatide (n = 751)	GLP-1RA (n = 751)	Risk Difference (95% CI)	ARR (%)	RRR (%)	HR (95% CI)	*P* Value	E Value for HR	E Value for Lower CI of HR
Primary outcome									
Composite of acute myocardial infarction, ischemic stroke, and all-cause mortality	54	90	−0.048 (−0.078 to −0.018)	5	40%	0.600 (0.428-0.841)	0.003	2.72	4.1
Secondary outcomes									
Acute myocardial infarction	32	55	−0.031 (−0.054 to −0.007)	3	42%	0.589 (0.381-0.911)	0.016	2.79	4.69
Ischemic stroke	21	26	−0.007 (−0.024 to 0.011)	1	19%	0.809 (0.455-1.437)	0.468	1.78	3.82
All-cause mortality	10	18	−0.011 (−0.024 to 0.003)	1	44%	0.352 (0.140-0.888)	0.021	5.13	13.77
All-cause hospitalization or ER visits	310	332	−0.029 (−0.079 to 0.021)	3	7%	0.940 (0.805-1.097)	0.432	1.26	1.6
Heart failure exacerbation	27	45	−0.024 (−0.046 to −0.002)	2	40%	0.609 (0.378-0.982)	0.040	2.67	4.73
New systolic heart failure	74	100	−0.035 (−0.067 to −0.002)	3	26%	0.736 (0.545-0.994)	0.045	2.06	3.07
New onset atrial fibrillation/flutter	10	18	−0.013 (−0.030 to 0.003)	1	45%	0.233 (0.079-0.689)	0.004	8.05	24.81
Acute kidney injury	52	78	−0.035 (−0.063 to −0.006)	3	33%	0.676 (0.476-0.960)	0.028	2.32	3.62
Renal replacement therapy	0	10	−0.013 (−0.022 to −0.005)	1	NA	NA	NA	NA	NA
HbA1c ≤7%	422	324	0.130 (0.080 to 0.181)	−13	−30%	1.507 (1.303-1.743)	<0.001	1.99	1.69
CRP ≥5 mg/L	14	19	−0.009 (−0.028 to 0.009)	1	26%	0.761 (0.381-1.521)	0.438	1.96	4.69
LDL ≤70 mg/dL	277	232	0.060 (0.012-0.108)	−6	−19%	1.326 (1.113-1.579)	0.002	1.73	1.37
Triglyceride ≤150 mg/dL	293	254	0.052 (0.003-0.101)	−5	−15%	1.267 (1.070-1.499)	0.006	1.64	1.27
Albumin: creatinine ratio ≤30 mg/g	49	30	0.025 (0.003-0.048)	−3	−63%	1.757 (1.115-2.770)	0.014	2.91	1.47
Albumin: creatinine ratio ≤300 mg/g	70	48	0.029 (0.002-0.056)	−3	−46%	1.561 (1.080-2.255)	0.017	2.5	1.37
BMI ≤30 kg/m^2^	181	194	−0.017 (−0.061 to 0.026)	2	7%	0.931 (0.760-1.141)	0.492	1.28	1.71
Pulmonary hypertension	33	44	−0.015 (−0.037 to 0.008)	1	25%	0.767 (0.489-1.206)	0.249	1.93	3.51

ARR = absolute risk reduction; CRP = C-reactive protein; GLP-1RA = glucagon-like peptide-1 receptor agonist; HbA1c = hemoglobin A1C; LDL = low-density lipoprotein; RRR = relative risk reduction.

a After propensity score matching.

#### Safety outcomes

The incidence of GI symptoms (HR: 0.68 [95% CI: 0.56-0.83], *P* < 0.001), palpitations (HR: 0.74 [95% CI: 0.54-1.00], *P* = 0.002), and influenza/pneumonia (HR: 0.58 [95% CI: 0.37-0.92], *P* = 0.021) were less in the tirzepatide group than in the GLP-1RA group. However, gallbladder and pancreatic disorders (HR: 0.81 [95% CI: 0.35-1.85], *P* = 0.620), NAFLD/hepatic fibrosis (HR: 0.89 [95% CI: 0.63-1.25], *P* = 0.513), and diabetic retinopathy (HR: 0.83 [95% CI: 0.59-1.17], *P* = 0.306) were comparable in both groups (Table 3).

**Table 3:** Comparison of Safety Outcomes

	Tirzepatide (n = 751)	GLP-1RA (n = 751)	Risk Difference (95% CI)	HR (95% CI)	*P* Value	E Value for HR	E Value for Lower CI of HR
GI symptoms	167	238	−0.095 (−0.139 to −0.050)	0.687 (0.564-0.837)	<0.001	1.92	2.33
Hypoglycemia	10	10	0 (−0.012 to 0.012)	1.000	0.721	1.00	1.00
Gallbladder and Pancreas disorders	10	13	−0.004 (−0.019 to 0.010)	0.812 (0.356-1.852)	0.620	1.77	5.06
Palpitations	73	101	−0.037 (−0.070 to −0.005)	0.740 (0.547-1.000)	0.049	2.04	3.06
Diabetic retinopathy	61	73	−0.016 (−0.045 to 0.013)	0.837 (0.596-1.177)	0.306	1.68	2.74
Influenza and pneumonia	29	51	−0.029 (−0.052 to −0.007)	0.588 (0.373-0.929)	0.021	2.79	4.8
Suicidal ideation/attempt	10	10	0 (−0.012 to 0.012)	1.000	0.010	1.00	1.00
Thyroid cancer	10	10	0 (−0.012 to 0.012)	1.000	0.557	1.00	1.00

GI = gastrointestinal; GLP-1RA = glucagon-like peptide-1 receptor agonist.

#### Subgroup analysis

For subgroup analysis of tirzepatide vs semaglutide/liraglutide, the primary composite outcomes were seen favorbaly with tirzepatide (HR: 0.56, 95% CI: 0.40-0.78, *P* = 0.001), primarily driven by AMI (HR: 0.55, 95% CI: 0.36-0.84, *P* = 0.005) and all-cause mortality (HR: 0.34, 95% CI: 0.13-0.86, *P* = 0.01). Ischemic stroke events were less in adults on tirzepatide than in those on semaglutide/liraglutide but did not reach statistical significance (HR: 0.76, 95% CI: 0.43-1.34, *P* = 0.34). For secondary outcomes, HFE (HR: 0.53, 95% CI: 0.34-0.84, *P* = 0.007), new-onset atrial fibrillation, or atrial flutter (HR: 0.35, 95% CI: 0.13-0.98, *P* = 0.03) was less associated with tirzepatide, whereas new systolic heart failure (HR: 0.8, 95% CI: 0.65-1.22, *P* = 0.49) and new-onset AKI (HR: 0.80, 95% CI: 0.55-1.15, *P* = 0.22) were not statistically different in both groups.

### Sensitivity analyses

A “look back” 12-month healthcare utilization of tirzepatide vs GLP-1RA groups for outpatient and emergency room visits and hospitalizations before January 1, 2022 did not show any differences between the 2 cohorts at baseline. These are represented at baseline and after PSM in Table 1. Falsification outcomes of urinary tract infections, peptic ulcer disease, and ambulatory visits during the follow-up period (Table 4) showed no differences in tirzepatide vs GLP-1RA groups. Furthermore, we used E-value measurements, as shown in Tables 2 and 3, which suggests additional confounding to be of a lesser degree.

**Table 4:** Falsification End-points

	Tirzepatide (n = 751)	GLP-1RA (n = 751)	OR (95% CI)	*P* Value	HR (95% CI)	*P* Value
Urinary tract infections	43	62	0.675 (0.451-1.01)	0.364	1.042 (0.94-1.155)	0.423
Peptic ulcer disease	10	14	0.71 (0.314-1.61)	0.41	0.742 (0.329-1.672)	0.47
Ambulatory visits	736	731	1.394 (0.678-2.866)	0.06	1.042 (0.94-1.155)	0.423

GLP-1RA = glucagon-like peptide-1 receptor agonist.

## Discussion

While the cardiovascular outcomes trial comparing tirzepatide to GLP-1RA is still underway, our observational cohort study utilizing a large research network database demonstrates the following key findings: 1) in people living with with T2DM, age ≥40 years, BMI ≥25 kg/m^2^, and pre-existing IHD, tirzepatide was associated with a reduction in the primary composite endpoint of AMI, ischemic stroke, and all-cause mortality relative to GLP-1RA; 2) individual components of the primary composite endpoint including AMI and all-cause mortality, but not ischemic stroke, were significantly less frequent in the tirzepatide group. In addition, multiple secondary outcomes including HFE, new systolic heart failure, atrial arrhythmias, and new-onset AKI were also lower in the tirzepatide group; and 3) tirzepatide was associated with a greater improvement in several biomarkers such as HbA1c, LDL, triglycerides, and albumin:creatinine ratio compared to GLP-1RA. In the absence of RCT data, these real-world data-based findings suggest that GIP/GLP-1RA use may have a greater impact on certain cardiovascular and laboratory outcomes than GLP-1RA (Central Illustration).

**Central Illustration:**
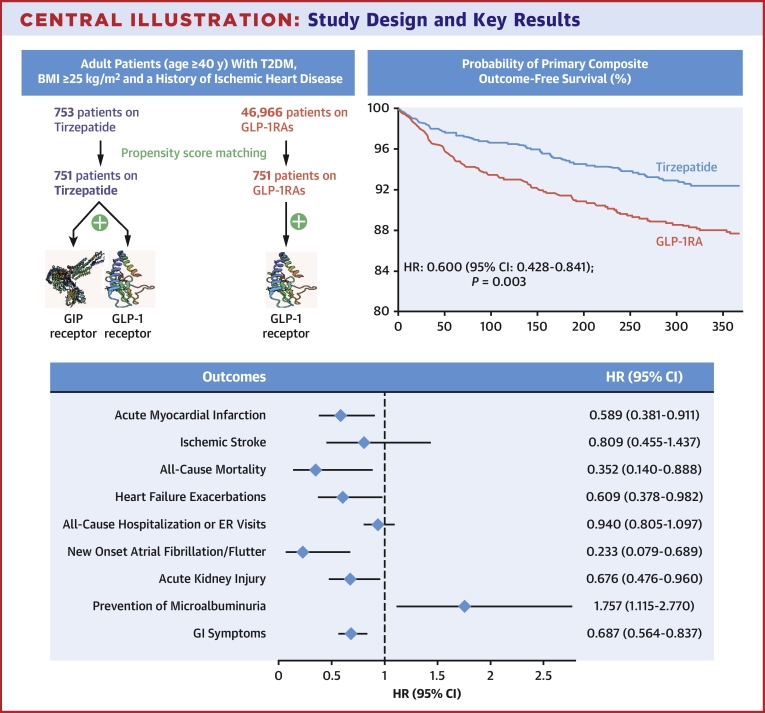
Study Design and Key Results

Recently, a much-needed paradigm shift has occurred in the management of T2DM with a focus on early intervention and intensive management of Cardiac-Renal-Metabolic (CaReMe) diseases including atherosclerotic CVD, heart failure, hypertension, dyslipidemia, atrial fibrillation, pre-diabetes, CKD, and NAFLD.^23^^,^^24^ As a result, the search for an ideal pharmacotherapeutic agent targeting multiple receptor pathways has intensified. While oral SGLT-2i have significant benefits with regards to glycemic control, heart failure hospitalizations and mortality, and reno-protection, injectable GLP-1RA has taken a center stage in obesity management and cardiovascular protection and has shown improved quality of life in patients with heart failure with preserved ejection fraction and obesity.^23^^,^^25^, ^26^, ^27^, ^28^ The impetus for developing the dual GIP/GLP-1RA agonist, tirzepatide, was found in postbariatric surgery patients who were noted to have elevated levels of GIP/GLP-1.^29^ Whether the benefits of weight loss, glycemic control, lipid lowering, reduced inflammation, and blood pressure control with GIP/GLP-1RA agonism translate to improved cardiovascular outcomes has not yet been demonstrated in RCTs or real-world data. Thus, our analysis adds clinically meaningful data to the literature regarding the potential cardiovascular benefits of GIP/GLP-1 agonists.

Tirzepatide was approved for the treatment of T2DM in 2022 and for the treatment of obesity in 2023. Using real-world data and propensity matching to control for potential confounders, our analysis shows that tirzepatide improved cardiovascular outcomes compared to GLP-1RA over a 12-month follow-up period. In particular, we noted a significant reduction in AMI (32 vs 55 events, HR: 0.58) and all-cause mortality (10 vs 18 events, HR: 0.35) relative to GLP-1RA. Reduction in ischemic stroke was found to be similar with tirzepatide vs GLP-1RA. Our results agree with a prior prespecified meta-analysis of the SURPASS trials^19^ which showed no change in stroke events with tirzepatide vs control groups. The SURPASS-4 trial evaluated cardiovascular outcomes with tirzepatide^30^ and demonstrated a reduction in MACE with HR: 0.50 (95% CI: 0.26-0.95). However, there were only 11 MACE in this trial, making it underpowered to detect statistical significance.

In parallel with prior studies of SGLT-2i and GLP-1RAs on HF outcomes, we examined HFE and new systolic heart failure in both groups. Tirzepatide was associated with a lower incidence of HFE (27 vs 45 events, HR: 0.60) and new systolic heart failure (74 vs 100 events, HR: 0.73). These findings are significant for future applications of GIP/GLP-1RA. Whether the effects represent an additive benefit of GIP agonism on natriuresis or tirzepatide-induced attenuation of lipopolysaccharide-induced left ventricular remodeling and dysfunction by inhibiting the TLR4/NF-kB/NLRP3 pathway^31^ needs to be explored. The SUMMIT trial (NCT04847557)^32^ will explore the role of tirzepatide in heart failure with preserved ejection fraction and obesity.

Tirzepatide's effect on atrial arrhythmias has been explored in a metanalysis of the SURPASS trials. In pooled data from SURPASS 2 to 5, the risk of AF (risk ratio = 1.59, 95% CI: 0.46-5.47, *P* = 0.47) was similar with tirzepatide compared to placebo or an active comparator.^32^ In our analysis, we found fewer new atrial fibrillation/flutter events (10 vs 18, HR: 0.23), favouring tirzepatide. Whether such effects are a result of weight loss, better blood pressure control, or speculative reduced atrial remodelling due to anti-inflammatory effects or agonism of GIP/GLP-1 receptors expressed on epicardial adipose tissue^33^ remains to be explored.

In the SURPASS-4 trial,^30^ tirzepatide use was associated with a slower decline in eGFR, decreased albuminuria, and a significantly reduced occurrence of the composite renal endpoint of eGFR decline ≥40% from baseline, end-stage kidney disease, death due to kidney failure, or new-onset macroalbuminuria (HR: 0.58, 95% CI: 0.43-0.80). Significant reduction in albuminuria was also found in a recently published metanalysis of 9,533 patients pooled from 8 RCTs.^34^ Albuminuria is directly proportional to cardiorenal outcomes, and these findings raise questions about the potential role of GIP/GLP-1RA in the renal endothelium and peri-renal adipose tissue. A few observational and pharmacovigilance studies had raised questions about the increased risk of AKI with GLP-1RA^35^^,^^36^ which later resolved in metanalysis specifically looking at AKI as an outcome. Our analysis demonstrated a lower incidence of new-onset AKI with tirzepatide than GLP-1RAs, further necessitating a need to study acute kidney outcomes in RCTs.

Finally, weight loss with tirzepatide vs GLP-1RA was found to be comparable in our analysis. We used occurrence of BMI <30 kg/m^2^ as a surrogate marker for weight loss during the follow-up period as only aggregate-level patient data were available in the database. Multiple studies have reported significant dose-dependent weight loss with tirzepatide; however, we could not assess such outcomes in the present analysis. Future studies should evaluate whether BMI, a crude way of measuring body fat, vs measurement of body adipose index utilizing dual-energy X-ray absorptiometry is the best way to assess the effects of these novel weight loss agents.^37^ GI symptoms, palpitations, influenza, and pneumonia were associated with GLP-1RA more than tirzepatide, whereas gall bladder, pancreatic disorders, hepatic fibrosis, and diabetic retinopathy were comparable. Continued surveillance and pharmacovigilance is needed to identify any adverse event signals as the uptake and adoption of tirzepatide increases in clinical practice.

### Study Limitations

Data in this study were extracted from the aggregate EHR database (TriNetX) and, therefore, may not contain accurately reported health conditions or symptomatology and does not capture outcomes occurring outside this database. We selected patients based on ICD-10 and Current Procedural Terminology coding; thus, our data are subject to the appropriate entry of such codes. However, both cohorts would be affected similarly by this process. In addition, we did not do a “look-back” to see the timelines of IHD development in these patients. This may have resulted in an unequal distribution of sicker individuals in the GLP-1RA vs tirzepatide groups, inducing selection bias. While we emulated the inclusion/exclusion criteria of the SURPASS-CVOT trial, it is crucial to note that individual patient-level data were not available, and thus, various BMI categories, improvement of BMI, the extent of weight loss, and data on other individual-level confounding variables were not available. Similarly, patients' blood pressure values at baseline and after initiating tirzepatide or GLP-1RA were unavailable. The database did not allow for the extraction of dosage information or dose escalation, and analysis based on dosing was not possible. Furthermore, cardiovascular mortality could not be differentiated from all-cause mortality in our database.

The results pertaining to all-cause mortality benefits appear larger than those usually found in RCTs. However, given the limitations of real-world data and despite PSM and sensitivity analyses, we cannot eliminate the possibility of selection, treatment attribution, immortal time bias, and unmeasured confounding biases due to significant social determinants of health. Our study used a 20-year retrieval window for baseline characteristics, which may have captured outdated medications or comorbidities that may not reflect the current clinical status of patients. Due to the query design of our study, people who have already been using tirzepatide or GLP-1RA for some time might have been included, rather than “incident” new users, and hence the possibility of prevalent user bias cannot be ruled out. Despite this, the practical implications of outcomes with the use of tirzepatide should remain largely unaffected. We acknowledge that 1:2 or 1:3 matching could have improved precision as there was substantial variation in patients on GLP-1RA vs tirzepatide before PSM. However, the limitations of the TrinetX analytics only allowed 1:1 matching.

While we did not restrict GLP-1RA to semaglutide or liraglutide in our analysis, the number of patients on older GLP-1RAs was small. In subgroup analyses, the results of tirzepatide vs liraglutide/semaglutide were similar to the principal analysis.

To account for measured and unmeasured biases, we evaluated baseline healthcare utilization in the form of all-cause hospitalization and ER visits within the prior 12 months to better match the population. In addition, we assessed for falsification of outcomes in the form of urinary tract infections, peptic ulcer disease, and ambulatory visits in the same follow-up time frame and found that this was similar between the 2 cohorts. We also performed the E-value calculation as a sensitivity analysis, a measure to check for robustness against bias from unmeasured confounding or omitted covariates in observational studies for both primary and secondary outcomes. A high E-value implies that a stronger unmeasured confounder would be needed to negate the covariate effect estimate and increase the likelihood of causality.

## Conclusions

In summary, our real-world analysis suggests that in people living with T2DM, age ≥40 years, overweight or obesity (BMI ≥25 kg/m^2^), and pre-existing IHD, treatment with tirzepatide vs GLP-1RA was associated with a lower risk of the composite endpoint of AMI, stroke and all-cause mortality. Tirzepatide was also associated with a lower incidence of HFE, new systolic heart failure, atrial arrhythmias, and AKI. In addition, tirzepatide was associated with a greater improvement in biochemical markers, including HbA1c, LDL, triglycerides, and albuminuria, while maintaining a comparable safety profile to GLP-1RA. These real-world data should reassure clinicians prescribing tirzepatide while we await the results of the SURPASS-CVOT trial evaluating cardiovascular outcomes with this agent.

Perspectives **COMPETENCY IN MEDICAL KNOWLEDGE OR PATIENT CARE:** In obese adults, older than 40 years, with T2DM, and prior ischemic heart disease, treatment with tirzepatide was associated with a lower risk of the combined risk of acute myocardial infarction, stroke, and all-cause mortality than GLP-1 agonists. As pharmacotherapies for the management of T2DM and obesity expand, a better understanding of the impact of these medications on cardiovascular outcomes will inform clinical practice.

**TRANSLATIONAL OUTLOOK:** Prospective, randomized clinical trials are needed to evaluate the impact of tirzepatide vs GLP-1 agonists on cardiovascular outcomes in obese adults with T2DM and pre-existing ischemic heart disease.

## Funding support and author disclosures

Dr Fonarow reports having consulted for Abbott, Amgen, AstraZeneca, Bayer, Boehringer Ingelheim, Cytokinetics, Eli Lilly, Johnson & Johnson, Medtronic, Merck, Novartis, and Pfizer. Dr Bhatt discloses the following relationships: is in the advisory board of Angiowave, Bayer, Boehringer Ingelheim, CellProthera, Cereno Scientific, Elsevier Practice Update Cardiology, High Enroll, Janssen, Level Ex, McKinsey, Medscape Cardiology, Merck, MyoKardia, NirvaMed, Novo Nordisk, PhaseBio, PLx Pharma, and Stasys; is a part of Board of Directors at American Heart Association New York City, Angiowave (stock options), Bristol Myers Squibb (stock), DRS.LINQ (stock options), and High Enroll (stock); is a consultant for Broadview Ventures, GlaxoSmithKline, Hims, SFJ, and Youngene; is a part of Data Monitoring Committees at Acesion Pharma, Assistance Publique-Hôpitaux de Paris, Baim Institute for Clinical Research (formerly Harvard Clinical Research Institute, for the PORTICO trial, funded by 10.13039/100006279St. Jude Medical, now Abbott), Boston Scientific (Chair, PEITHO trial), Cleveland Clinic, Contego Medical (Chair, PERFORMANCE 2), Duke Clinical Research Institute, Mayo Clinic, Mount Sinai School of Medicine (for the ENVISAGE trial, funded by 10.13039/501100002973Daiichi Sankyo; for the ABILITY-DM trial, funded by Concept Medical; for ALLAY-HF, funded by Alleviant Medical), Novartis, Population Health Research Institute; Rutgers University (for the NIH-funded MINT Trial); received Honoraria from American College of Cardiology (Senior Associate Editor, Clinical Trials and News, ACC.org; is the Chair of the ACC Accreditation Oversight Committee), Arnold and Porter law firm (work related to Sanofi/Bristol-Myers Squibb clopidogrel litigation), Baim Institute for Clinical Research (formerly Harvard Clinical Research Institute; RE-DUAL PCI clinical trial steering committee funded by 10.13039/100001003Boehringer Ingelheim; AEGIS-II executive committee funded by 10.13039/100008322CSL Behring), Belvoir Publications (Editor in Chief, Harvard Heart Letter), Canadian Medical and Surgical Knowledge Translation Research Group (clinical trial steering committees), CSL Behring (AHA lecture), Cowen and Company, Duke Clinical Research Institute (clinical trial steering committees, including for the PRONOUNCE trial, funded by Ferring Pharmaceuticals), HMP Global (Editor in Chief, *Journal of Invasive Cardiology*), *Journal of the American College of Cardiology* (Guest Editor; Associate Editor), K2P (Co-Chair, interdisciplinary curriculum), Level Ex, Medtelligence/ReachMD (CME steering committees), MJH Life Sciences, Oakstone CME (Course Director, Comprehensive Review of Interventional Cardiology), Piper Sandler, Population Health Research Institute (for the COMPASS operations committee, publications committee, steering committee, and USA national co-leader, funded by Bayer), WebMD (CME steering committees), Wiley (steering committee); other: Clinical Cardiology (Deputy Editor); Patent: Sotagliflozin (named on a patent for sotagliflozin assigned to Brigham and Women's Hospital who assigned to Lexicon; neither I nor Brigham and Women's Hospital receive any income from this patent); Research Funding: Abbott, Acesion Pharma, Afimmune, Aker Biomarine, Alnylam, Amarin, Amgen, AstraZeneca, Bayer, Beren, Boehringer Ingelheim, Boston Scientific, Bristol-Myers Squibb, Cardax, CellProthera, Cereno Scientific, Chiesi, CinCor, Cleerly, CSL Behring, Eisai, Ethicon, Faraday Pharmaceuticals, Ferring Pharmaceuticals, Forest Laboratories, Fractyl, Garmin, HLS Therapeutics, Idorsia, Ironwood, Ischemix, Janssen, Javelin, Lexicon, Lilly, Medtronic, Merck, Moderna, MyoKardia, NirvaMed, Novartis, Novo Nordisk, Otsuka, Owkin, Pfizer, PhaseBio, PLx Pharma, Recardio, Regeneron, Reid Hoffman Foundation, Roche, Sanofi, Stasys, Synaptic, The Medicines Company, Youngene, 89Bio; Royalties: Elsevier (Editor, Braunwald's Heart Disease); Site Co-Investigator: Abbott, Biotronik, Boston Scientific, CSI, Endotronix, St. Jude Medical (now Abbott), Philips, SpectraWAVE, Svelte, Vascular Solutions; Trustee: American College of Cardiology; Unfunded Research: FlowCo. Dr Jhund reports speaker fees from AstraZeneca, Novartis, Alkem Metabolics, ProAdWise Communications, Sun Pharmaceuticals, and Intas Pharmaceuticals; advisory board fees from AstraZeneca, Boehringer Ingelheim, and Novartis; research funding from 10.13039/100004325AstraZeneca, 10.13039/100001003Boehringer Ingelheim, and Analog Devices Inc; and is the director of Global Clinical Trial Partners. Dr Jhund's employer, the University of Glasgow, has been remunerated for clinical trial work from AstraZeneca, Bayer AG, Novartis, and Novo Nordisk. Dr Kosiborod has received research grants from 10.13039/100004325AstraZeneca and Boehringer Ingelheim and has served as a consultant for Alnylam, AstraZeneca, Amgen, Applied Therapeutics, Bayer, Boehringer Ingelheim, Cytokinetics, Eli Lilly, Esperion Therapeutics, Janssen, Merck (Diabetes and Cardiovascular), Novo Nordisk, Pharmacosmos, Sanofi, and Vifor. All other authors have reported that they have no relationships relevant to the contents of this paper to disclose.
