# Supplementary material for: An Observational Study of Cardiovascular Outcomes of Tirzepatide vs Glucagon-Like Peptide-1 Receptor Agonists
Source: JACC Adv. 2025 May 28;4(5):101740. doi: 10.1016/j.jacadv.2025.101740 (PMC12235410; doi:10.1016/j.jacadv.2025.101740)

**Supplemental Appendix for**

**An Observational Study of Cardiovascular Outcomes of Tirzepatide vs. Glucagon-Like Peptide-1 receptor agonists (GLP-1RA)**

Contents

[Cohorts definition 2](#_Toc187746889)

[Supplementary Table 1: Query Criteria for Cohort 1 (query name: Age>=40, Tirzepatide, BMI>=25, IHD) 2](#_Toc187746890)

[Supplementary Table 2. Query Criteria for Cohort 2 (query name: Age>=40, GLP, BMI>=25, IHD) 2](#_Toc187746891)

[Supplementary Table 3. Outcome Definitions 3](#_Toc187746892)

[Supplementary Table 4. Propensity Score Matching 9](#_Toc187746893)

[Supplementary Table 5. Baseline Covariates Matched in PSM and their Standardized Codes in the TriNetX Database 10](#_Toc187746894)

[Supplementary Table 6. Survival Probabilities for Primary Composite Outcomes and Heart Failure Exacerbation 12](#_Toc187746895)

[Supplementary Figure 1. Consort Diagram 13](#_Toc187746896)

# Cohorts definition

This section lists all terms used in the definitions of the two cohorts.

# Supplementary Table 1: Query Criteria for Cohort 1 (query name: Age>=40, Tirzepatide, BMI>=25, IHD)

This query was run on the network Research with 82 HCO(s) queried and 82 HCO(s) responded. A total of 14 provider(s) responded with patients. The final cohort included 753 patients who matched the query criteria listed in the table below. For the text representation of the query criteria please see Appendix A.

|  | | | | | |
| --- | --- | --- | --- | --- | --- |
| Ungrouped terms | | | | | |
|  | must have |  | demographics | Age | Age (at least 40 years (most recent occurrence)) |
| Group 1 | | | | | |
|  | **Group 1A** | | | | |
|  | must have |  | medication | NLM:RXNORM:2601723 | tirzepatide |
|  | date constraint | | The terms in this group occurred after Jan 1, 2022 and before Dec 31, 2022 | | |
|  | event relationship | | Any instance of Group 1B occurred at least 2 months before any instance of Group 1A | | |
|  | **Group 1B** | | | | |
|  | must have | any of | laboratory | TNX:9083 | BMI (at least 25.00 kg/m2) |
|  |  |  | laboratory | UMLS:LNC:39156-5 | Body Mass Index (at least 25.00 kg/m2) |
|  |  | and | diagnosis | UMLS:ICD10CM:E11 | Type 2 diabetes mellitus |
|  |  | and | diagnosis | UMLS:ICD10CM:I20-I25 | Ischemic heart diseases |

# Supplementary Table 2. Query Criteria for Cohort 2 (query name: Age>=40, GLP, BMI>=25, IHD)

This query was run on the network Research with 82 HCO(s) queried and 82 HCO(s) responded. A total of 24 provider(s) responded with patients. The final cohort included 46,967 patients who matched the query criteria listed in the table below.

| Ungrouped terms | | | | | |
| --- | --- | --- | --- | --- | --- |
|  | must have |  | demographics | Age | Age (at least 40 years (most recent occurrence)) |
| Group 1 | | | | | |
|  | **Group 1A** | | | | |
|  | must have | any of | medication | NLM:RXNORM:1991302 | semaglutide |
|  |  |  | medication | NLM:RXNORM:475968 | liraglutide |
|  |  |  | medication | NLM:RXNORM:1551291 | dulaglutide |
|  |  |  | medication | NLM:RXNORM:1440051 | lixisenatide |
|  |  |  | medication | NLM:RXNORM:60548 | exenatide |
|  | date constraint | | The terms in this group occurred after Jan 1, 2022 and before Dec 31, 2022 | | |
|  | event relationship | | Any instance of Group 1B occurred at least 2 months before any instance of Group 1A | | |
|  | **Group 1B** | | | | |
|  | must have | any of | laboratory | TNX:9083 | BMI (at least 25.00 kg/m2) |
|  |  |  | laboratory | UMLS:LNC:39156-5 | Body Mass Index (at least 25.00 kg/m2) |
|  |  | and | diagnosis | UMLS:ICD10CM:E11 | Type 2 diabetes mellitus |
|  |  | and | diagnosis | UMLS:ICD10CM:I20-I25 | Ischemic heart diseases |

# Supplementary Table 3. Outcome Definitions

Table below outlines the definitions for each outcome and the analysis specifications. For outcome definitions consisting of more than one term, at least one term must match. Please see Appendix C for the text representation of the outcome definitions.

| Primary Composite Outcome: AMI/Stroke/All cause mortality | | | | |
| --- | --- | --- | --- | --- |
|  | **Outcome definition** | | | |
|  | | Diagnosis | UMLS:ICD10CM:I21 | Acute myocardial infarction |
|  | | Diagnosis | UMLS:ICD10CM:I63 | Cerebral infarction |
|  | | Demographics | Deceased | Deceased |
|  | **Settings for the performed analyses** | | | |
|  | | Risk analysis | | including patients with outcome prior to the time window |
|  | | Kaplan - Meier survival analysis | | including patients with outcome prior to the time window |
|  | | Number of instances analysis | | including patients with outcome prior to the time window excluding patients with zero outcomes counts are grouped by date |
| Ischemic Stroke | | | | |
|  | **Outcome definition** | | | |
|  | | Diagnosis | UMLS:ICD10CM:I63 | Cerebral infarction |
|  | **Settings for the performed analyses** | | | |
|  | | Risk analysis | | including patients with outcome prior to the time window |
|  | | Kaplan - Meier survival analysis | | including patients with outcome prior to the time window |
|  | | Number of instances analysis | | including patients with outcome prior to the time window excluding patients with zero outcomes counts are grouped by date |
| AMI | | | | |
|  | **Outcome definition** | | | |
|  | | Diagnosis | UMLS:ICD10CM:I21 | Acute myocardial infarction |
|  | **Settings for the performed analyses** | | | |
|  | | Risk analysis | | including patients with outcome prior to the time window |
|  | | Kaplan - Meier survival analysis | | including patients with outcome prior to the time window |
|  | | Number of instances analysis | | including patients with outcome prior to the time window excluding patients with zero outcomes counts are grouped by date |

| All cause mortality | | | | |
| --- | --- | --- | --- | --- |
|  | **Outcome definition** | | | |
|  | | Demographics | Deceased | Deceased |
|  | **Settings for the performed analyses** | | | |
|  | | Risk analysis | | including patients with outcome prior to the time window |
|  | | Kaplan - Meier survival analysis | | including patients with outcome prior to the time window |
|  | | Number of instances analysis | | including patients with outcome prior to the time window excluding patients with zero outcomes counts are grouped by date |

| HF Exacerbation | | | | |
| --- | --- | --- | --- | --- |
|  | **Outcome definition** | | | |
|  | | Medication | NLM:VA:CV702 | LOOP DIURETICS (Route: Injectable Product) |
|  | | Medication | NLM:RXNORM:6916 | metolazone |
|  | | Medication | NLM:RXNORM:2396 | chlorothiazide (Route: Injectable Product) |
|  | | Diagnosis | UMLS:ICD10CM:J81 | Pulmonary edema |
|  | **Settings for the performed analyses** | | | |
|  | | Risk analysis | | including patients with outcome prior to the time window |
|  | | Kaplan - Meier survival analysis | | including patients with outcome prior to the time window |
|  | | Number of instances analysis | | including patients with outcome prior to the time window excluding patients with zero outcomes counts are grouped by date |
| All cause hospitalization or ER visits | | | | |
|  | **Outcome definition** | | | |
|  | | Visit | UMLS:HL7V3.0:VisitType:EMER | Visit: Emergency |
|  | | Visit | UMLS:HL7V3.0:VisitType:ACUTE | Visit: Inpatient Acute |
|  | | Visit | UMLS:HL7V3.0:VisitType:IMP | Visit: Inpatient Encounter |
|  | | Visit | UMLS:HL7V3.0:VisitType:NONAC | Visit: Inpatient Non-acute |
|  | | Visit | UMLS:HL7V3.0:VisitType:OBSENC | Visit: Observation Encounter |
|  | | Visit | UMLS:HL7V3.0:VisitType:SS | Visit: Short Stay |
|  | **Settings for the performed analyses** | | | |
|  | | Risk analysis | | including patients with outcome prior to the time window |
|  | | Kaplan - Meier survival analysis | | including patients with outcome prior to the time window |
|  | | Number of instances analysis | | including patients with outcome prior to the time window excluding patients with zero outcomes counts are grouped by date |
| AFib/Aflutter | | | | |
|  | **Outcome definition** | | | |
|  | | Diagnosis | UMLS:ICD10CM:I48 | Atrial fibrillation and flutter |
|  | **Settings for the performed analyses** | | | |
|  | | Number of instances analysis | | excluding patients with outcome prior to the time window excluding patients with zero outcomes counts are grouped by date |
|  | | Kaplan - Meier survival analysis | | excluding patients with outcome prior to the time window |
|  | | Risk analysis | | excluding patients with outcome prior to the time window |
| Renal replacement therapy | | | | |
|  | **Outcome definition** | | | |
|  | | Procedure | UMLS:CPT:1012740 | Dialysis Services and Procedures |
|  | **Settings for the performed analyses** | | | |
|  | | Number of instances analysis | | excluding patients with outcome prior to the time window excluding patients with zero outcomes counts are grouped by date |
|  | | Kaplan - Meier survival analysis | | excluding patients with outcome prior to the time window |
|  | | Risk analysis | | excluding patients with outcome prior to the time window |
| CRP>=5 | | | | |
|  | **Outcome definition** | | | |
|  | | Laboratory | TNX:9063 | C reactive protein [Mass/volume] in Serum, Plasma or Blood (at least 5.00 mg/L (most recent occurrence)) |
|  | **Settings for the performed analyses** | | | |
|  | | Risk analysis | | excluding patients with outcome prior to the time window |
|  | | Kaplan - Meier survival analysis | | excluding patients with outcome prior to the time window |
|  | | Number of instances analysis | | excluding patients with outcome prior to the time window excluding patients with zero outcomes counts are grouped by date |
| Pulmonary Hypertension | | | | |
|  | **Outcome definition** | | | |
|  | | Diagnosis | UMLS:ICD10CM:I27.0 | Primary pulmonary hypertension |
|  | | Diagnosis | UMLS:ICD10CM:I27.2 | Other secondary pulmonary hypertension |
|  | **Settings for the performed analyses** | | | |
|  | | Kaplan - Meier survival analysis | | including patients with outcome prior to the time window |
|  | | Risk analysis | | including patients with outcome prior to the time window |
|  | | Number of instances analysis | | including patients with outcome prior to the time window excluding patients with zero outcomes counts are grouped by date |
| Malignancy | | | | |
|  | **Outcome definition** | | | |
|  | | GlobalOncology | UMLS:ICDO3:C00-C80 | Oncology: Primary Site |
|  | **Settings for the performed analyses** | | | |
|  | | Number of instances analysis | | excluding patients with outcome prior to the time window excluding patients with zero outcomes counts are grouped by date |
|  | | Kaplan - Meier survival analysis | | excluding patients with outcome prior to the time window |
|  | | Risk analysis | | excluding patients with outcome prior to the time window |
| AKI | | | | |
|  | **Outcome definition** | | | |
|  | | Diagnosis | UMLS:ICD10CM:N17 | Acute kidney failure |
|  | **Settings for the performed analyses** | | | |
|  | | Risk analysis | | including patients with outcome prior to the time window |
|  | | Kaplan - Meier survival analysis | | including patients with outcome prior to the time window |
|  | | Number of instances analysis | | including patients with outcome prior to the time window excluding patients with zero outcomes counts are grouped by date |
| Immunization | | | | |
|  | **Outcome definition** | | | |
|  | | Procedure | UMLS:CPT:1012589 | Immunization Administration for Vaccines/Toxoids |
|  | | Procedure | UMLS:CPT:1012602 | Vaccines, Toxoids |
|  | **Settings for the performed analyses** | | | |
|  | | Risk analysis | | including patients with outcome prior to the time window |
|  | | Kaplan - Meier survival analysis | | including patients with outcome prior to the time window |
|  | | Number of instances analysis | | including patients with outcome prior to the time window excluding patients with zero outcomes counts are grouped by date |
| GI Symptoms | | | | |
|  | **Outcome definition** | | | |
|  | | Diagnosis | UMLS:ICD10CM:R10-R19 | Symptoms and signs involving the digestive system and abdomen |
|  | **Settings for the performed analyses** | | | |
|  | | Risk analysis | | including patients with outcome prior to the time window |
|  | | Kaplan - Meier survival analysis | | including patients with outcome prior to the time window |
|  | | Number of instances analysis | | including patients with outcome prior to the time window excluding patients with zero outcomes counts are grouped by date |
| Palpitation | | | | |
|  | **Outcome definition** | | | |
|  | | Diagnosis | UMLS:ICD10CM:R00 | Abnormalities of heart beat |
|  | **Settings for the performed analyses** | | | |
|  | | Risk analysis | | including patients with outcome prior to the time window |
|  | | Kaplan - Meier survival analysis | | including patients with outcome prior to the time window |
|  | | Number of instances analysis | | including patients with outcome prior to the time window excluding patients with zero outcomes counts are grouped by date |
| GB and Pancreas Disorders | | | | |
|  | **Outcome definition** | | | |
|  | | Diagnosis | UMLS:ICD10CM:K80-K87 | Disorders of gallbladder, biliary tract and pancreas |
|  | **Settings for the performed analyses** | | | |
|  | | Risk analysis | | excluding patients with outcome prior to the time window |
|  | | Kaplan - Meier survival analysis | | excluding patients with outcome prior to the time window |
|  | | Number of instances analysis | | excluding patients with outcome prior to the time window excluding patients with zero outcomes counts are grouped by date |
| Thyroid cancer | | | | |
|  | **Outcome definition** | | | |
|  | | Diagnosis | UMLS:ICD10CM:C73 | Malignant neoplasm of thyroid gland |
|  | **Settings for the performed analyses** | | | |
|  | | Risk analysis | | including patients with outcome prior to the time window |
|  | | Kaplan - Meier survival analysis | | including patients with outcome prior to the time window |
|  | | Number of instances analysis | | including patients with outcome prior to the time window excluding patients with zero outcomes counts are grouped by date |
| Hypoglycemia | | | | |
|  | **Outcome definition** | | | |
|  | | Diagnosis | UMLS:ICD10CM:E16.0 | Drug-induced hypoglycemia without coma |
|  | | Diagnosis | UMLS:ICD10CM:E16.1 | Other hypoglycemia |
|  | | Diagnosis | UMLS:ICD10CM:E16.2 | Hypoglycemia, unspecified |
|  | **Settings for the performed analyses** | | | |
|  | | Risk analysis | | including patients with outcome prior to the time window |
|  | | Kaplan - Meier survival analysis | | including patients with outcome prior to the time window |
|  | | Number of instances analysis | | including patients with outcome prior to the time window excluding patients with zero outcomes counts are grouped by date |
| Diabetic retinopathy | | | | |
|  | **Outcome definition** | | | |
|  | | Diagnosis | UMLS:ICD10CM:E11.31 | Type 2 diabetes mellitus with unspecified diabetic retinopathy |
|  | | Diagnosis | UMLS:ICD10CM:E11.32 | Type 2 diabetes mellitus with mild nonproliferative diabetic retinopathy |
|  | | Diagnosis | UMLS:ICD10CM:E11.33 | Type 2 diabetes mellitus with moderate nonproliferative diabetic retinopathy |
|  | | Diagnosis | UMLS:ICD10CM:E11.34 | Type 2 diabetes mellitus with severe nonproliferative diabetic retinopathy |
|  | | Diagnosis | UMLS:ICD10CM:E11.35 | Type 2 diabetes mellitus with proliferative diabetic retinopathy |
|  | **Settings for the performed analyses** | | | |
|  | | Risk analysis | | including patients with outcome prior to the time window |
|  | | Kaplan - Meier survival analysis | | including patients with outcome prior to the time window |
|  | | Number of instances analysis | | including patients with outcome prior to the time window excluding patients with zero outcomes counts are grouped by date |
| Suicidal ideation/attempt | | | | |
|  | **Outcome definition** | | | |
|  | | Diagnosis | UMLS:ICD10CM:R45.851 | Suicidal ideations |
|  | | Diagnosis | UMLS:ICD10CM:T14.91 | Suicide attempt |
|  | **Settings for the performed analyses** | | | |
|  | | Risk analysis | | including patients with outcome prior to the time window |
|  | | Kaplan - Meier survival analysis | | including patients with outcome prior to the time window |
|  | | Number of instances analysis | | including patients with outcome prior to the time window excluding patients with zero outcomes counts are grouped by date |
| BMI<=30 | | | | |
|  | **Outcome definition** | | | |
|  | | Laboratory | TNX:9083 | BMI (at most 30.00 kg/m2 (most recent occurrence)) |
|  | | Laboratory | UMLS:LNC:39156-5 | Body Mass Index (at most 30.00 kg/m2 (most recent occurrence)) |
|  | **Settings for the performed analyses** | | | |
|  | | Risk analysis | | including patients with outcome prior to the time window |
|  | | Kaplan - Meier survival analysis | | including patients with outcome prior to the time window |
|  | | Number of instances analysis | | including patients with outcome prior to the time window excluding patients with zero outcomes counts are grouped by date |
| HbA1c<=7 | | | | |
|  | **Outcome definition** | | | |
|  | | Laboratory | TNX:9037 | Hemoglobin A1c/Hemoglobin.total in Blood (at most 7.00 % (most recent occurrence)) |
|  | | Laboratory | UMLS:LNC:4548-4 | Hemoglobin A1c/Hemoglobin.total in Blood (at most 7.00 % (most recent occurrence)) |
|  | **Settings for the performed analyses** | | | |
|  | | Risk analysis | | including patients with outcome prior to the time window |
|  | | Kaplan - Meier survival analysis | | including patients with outcome prior to the time window |
|  | | Number of instances analysis | | including patients with outcome prior to the time window excluding patients with zero outcomes counts are grouped by date |
| LDL <= 70 mg/dL | | | | |
|  | **Outcome definition** | | | |
|  | | Laboratory | TNX:9002 | Cholesterol in LDL [Mass/volume] in Serum or Plasma (at most 70.00 mg/dL (most recent occurrence)) |
|  | **Settings for the performed analyses** | | | |
|  | | Risk analysis | | including patients with outcome prior to the time window |
|  | | Kaplan - Meier survival analysis | | including patients with outcome prior to the time window |
|  | | Number of instances analysis | | including patients with outcome prior to the time window excluding patients with zero outcomes counts are grouped by date |
| Albumin/Cr <=30 | | | | |
|  | **Outcome definition** | | | |
|  | | Laboratory | UMLS:LNC:9318-7 | Albumin/Creatinine [Mass Ratio] in Urine (at most 30.00 units (most recent occurrence)) |
|  | | Laboratory | TNX:LG34557-5 | Albumin/Creatinine [Mass ratio] in Urine (at most 30.00 units (most recent occurrence)) |
|  | **Settings for the performed analyses** | | | |
|  | | Risk analysis | | including patients with outcome prior to the time window |
|  | | Kaplan - Meier survival analysis | | including patients with outcome prior to the time window |
|  | | Number of instances analysis | | including patients with outcome prior to the time window excluding patients with zero outcomes counts are grouped by date |
| Albumin/Cr <= 300 | | | | |
|  | **Outcome definition** | | | |
|  | | Laboratory | UMLS:LNC:9318-7 | Albumin/Creatinine [Mass Ratio] in Urine (at most 300.00 units (most recent occurrence)) |
|  | | Laboratory | TNX:LG34557-5 | Albumin/Creatinine [Mass ratio] in Urine (at most 300.00 units (most recent occurrence)) |
|  | **Settings for the performed analyses** | | | |
|  | | Risk analysis | | including patients with outcome prior to the time window |
|  | | Kaplan - Meier survival analysis | | including patients with outcome prior to the time window |
|  | | Number of instances analysis | | including patients with outcome prior to the time window excluding patients with zero outcomes counts are grouped by date |
| Triglyceride <= 150 | | | | |
|  | **Outcome definition** | | | |
|  | | Laboratory | TNX:9004 | Triglyceride [Mass/volume] in Serum, Plasma or Blood (at most 150.00 mg/dL (most recent occurrence)) |
|  | | Laboratory | UMLS:LNC:2571-8 | Triglyceride [Mass/volume] in Serum or Plasma (at most 150.00 mg/dL (most recent occurrence)) |
|  | **Settings for the performed analyses** | | | |
|  | | Risk analysis | | including patients with outcome prior to the time window |
|  | | Kaplan - Meier survival analysis | | including patients with outcome prior to the time window |
|  | | Number of instances analysis | | including patients with outcome prior to the time window excluding patients with zero outcomes counts are grouped by date |
| Systolic HF diagnosis | | | | |
|  | **Outcome definition** | | | |
|  | | Diagnosis | UMLS:ICD10CM:I50.2 | Systolic (congestive) heart failure |
|  | | Diagnosis | UMLS:ICD10CM:I50.4 | Combined systolic (congestive) and diastolic (congestive) heart failure |
|  | **Settings for the performed analyses** | | | |
|  | | Risk analysis | | including patients with outcome prior to the time window |
|  | | Kaplan - Meier survival analysis | | including patients with outcome prior to the time window |
|  | | Number of instances analysis | | including patients with outcome prior to the time window excluding patients with zero outcomes counts are grouped by date |

# Supplementary Table 4. Propensity Score Matching

| **Cohort 1 and cohort 2 patient count before and after propensity score matching** | | | | | |
| --- | --- | --- | --- | --- | --- |
|  | | Cohort | Patient count before matching | | Patient count after matching |
|  | | 1 - Age>=40, Tirzepatide, BMI>=25 IHD | 753 | | 751 |
|  | | 2 - Age>=40, GLP, BMI>=25 IHD | 46,966 | | 751 |
|  | | Total for both cohorts | 47,719 | | 1,502 |
| **Propensity score density function - Before and after matching (cohort 1 - purple, cohort 2 - green)** | | | | | |
|  |  | 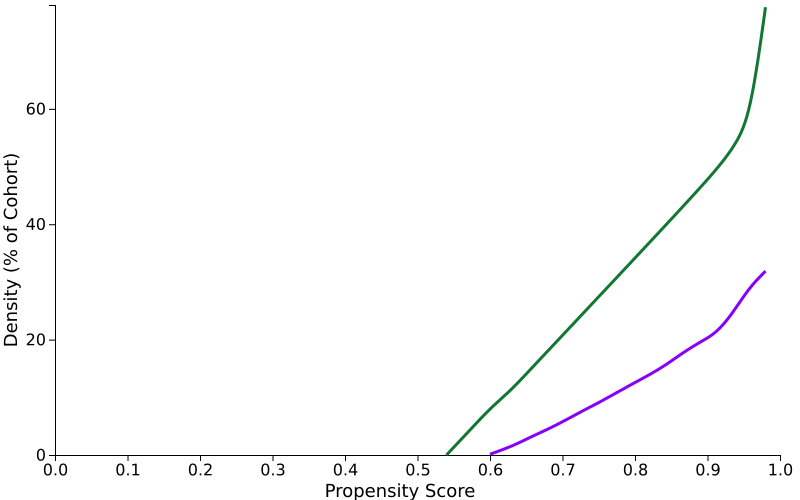 | | 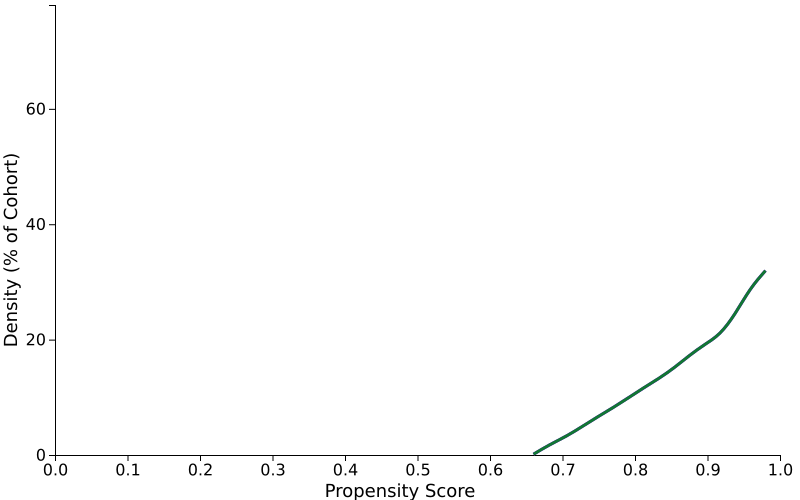 | |

# Supplementary Table 5. Baseline Covariates Matched in PSM and their Standardized Codes in the TriNetX Database

| Covariate | Code | Data Type |
| --- | --- | --- |
| **Demographics** | | |
| Age, years | AI | Continuous |
| Female | F | Yes/No |
| Non-Hispanic | 2186-5 | Yes/No |
| White | 2106-3 | Yes/No |
| BMI | 9083 | Continuous |
| **Comorbidities** | | |
| Hypertension | I10-I1A | Yes/No |
| Hyperlipidemia | E78 | Yes/No |
| Acute myocardial infarction | I21 | Yes/No |
| Ischemic stroke | I63 | Yes/No |
| History of prior PCI | 1021141 | Yes/No |
| Atrial fibrillation/flutter | I48 | Yes/No |
| Chronic kidney disease | N18 | Yes/No |
| Peripheral arterial disease | I70-I79 | Yes/No |
| Chronic lower respiratory diseases | J40-J4A | Yes/No |
| Malignancy | AN000 | Yes/No |
| **Medications** | | |
| Statin | CV350 | Yes/No |
| ACE-inhibitors | CV800 | Yes/No |
| ARB | CV805 | Yes/No |
| ARNi | 1656328 | Yes/No |
| Beta-blockers | CV100 | Yes/No |
| Antiarrhythmics | CV300 | Yes/No |
| Loop diuretics | CV702 | Yes/No |
| Thiazide diuretics | CV701 | Yes/No |
| Potassium-sparing diuretics | CV704 | Yes/No |
| Empagliflozin | 1545653 | Yes/No |
| Dapagliflozin | 1488564 | Yes/No |
| Canagliflozin | 1373458 | Yes/No |
| Aspirin | 1191 | Yes/No |
| Insulin | HS501 | Yes/No |
| Metformin | 6809 | Yes/No |
| Glipizide | 4821 | Yes/No |
| Clopidogrel | 32968 | Yes/No |
| Ticagrelor | 1116632 | Yes/No |
| Warfarin | 11289 | Yes/No |
| Apixaban | 1364430 | Yes/No |
| Rivaroxaban | 1114195 | Yes/No |
| Linagliptin | 1100699 | Yes/No |
| Saxagliptin | 857974 | Yes/No |
| Alogliptin | 1368001 | Yes/No |
| Sitagliptin | 593411 | Yes/No |
| **Lab Values** | | |
| Creatinine (mg/dL) | 9024 | Continuous |
| LVEF < 45% | 2003 | Yes/No |
| BNP > 150 pg/ml | 9003 | Yes/No |
| NT-proBNP > 450 pg/ml | 9072 | Yes/No |
| LDL Cholesterol > 130 mg/dL | 9002 | Yes/No |
| Triglyceride | 9004 | Continuous |
| Hemoglobin A1c ≥7% | 9037 | Yes/No |
| Aspartate aminotransferase | 9047 | Continuous |
| Alanine aminotransferase | 9044 | Continuous |
| CRP≥5 mg/L | 9063 | Yes/No |
| **Prior Healthcare Utilization** | | |
| PCI | 1021141 | Yes/No |
| Outpatient Visits | 1013626 | Yes/No |
| ER Visits | 1013711 | Yes/No |
| Inpatient admissions | 1013659 | Yes/No |
| **Abbreviations:** GLP-1RA: Glucagon-like peptide-1 receptor agonist; ACE: Angiotensin-converting enzyme; ARB: Angiotensin receptor blocker; ARNi: Angiotensin receptor neprilysin inhibitor; LDL: Low-density lipoprotein; BNP: Brain natriuretic peptide; CRP: C-Reactive protein; LVEF: Left Ventricular Ejection Fraction; PCI: Percutaneous Coronary Intervention | | |

# Supplementary Table 6. Survival Probabilities for Primary Composite Outcomes and Heart Failure Exacerbation

| **Outcome 1: Primary composite outcome of acute myocardial infarction, ischemic stroke, and all-cause mortality** | | |
| --- | --- | --- |
| **Time since index event (days)** | **Event-free survival probability and 95% CI with Tirzepatide (%)** | **Event-free survival probability and 95% CI with GLP-1RAs (%)** |
| 50 | 97.60 (96.21, 98.48) | 95.80 (94.00, 96.95) |
| 100 | 96.65 (95.08, 97.72) | 93.43 (91.40, 95.00) |
| 150 | 95.96 (94.27, 97.16) | 92.08 (89.89, 93.80) |
| 200 | 94.57 (92.66, 95.99) | 90.85 (88.53, 92.71) |
| 250 | 93.84 (91.84, 95.37) | 89.45 (87.01, 91.46) |
| 300 | 92.93 (90.80, 94.59) | 88.60 (86.08, 90.69) |
| 350 | 92.41 (90.20, 94.14) | 88.02 (85.44, 90.17) |
| **Outcome 2: Heart failure exacerbation** | | |
| 50 | 99.60 (98.76, 99.87) | 98.26 (97.02, 98.99) |
| 100 | 99.06 (98.04, 99.55) | 97.31 (95.87, 98.26) |
| 150 | 98.65 (97.50, 99.27) | 96.23 (94.58, 97.38) |
| 200 | 98.37 (97.14, 98.37) | 95.68 (93.94, 96.92) |
| 250 | 97.35 (95.87, 98.30) | 94.69 (92.81, 96.10) |
| 300 | 96.74 (95.13, 97.82) | 93.98 (92.00, 95.49) |
| 350 | 96.22 (94.49, 97.42) | 93.83 (91.82, 95.36) |

# Supplementary Figure 1. Consort Diagram


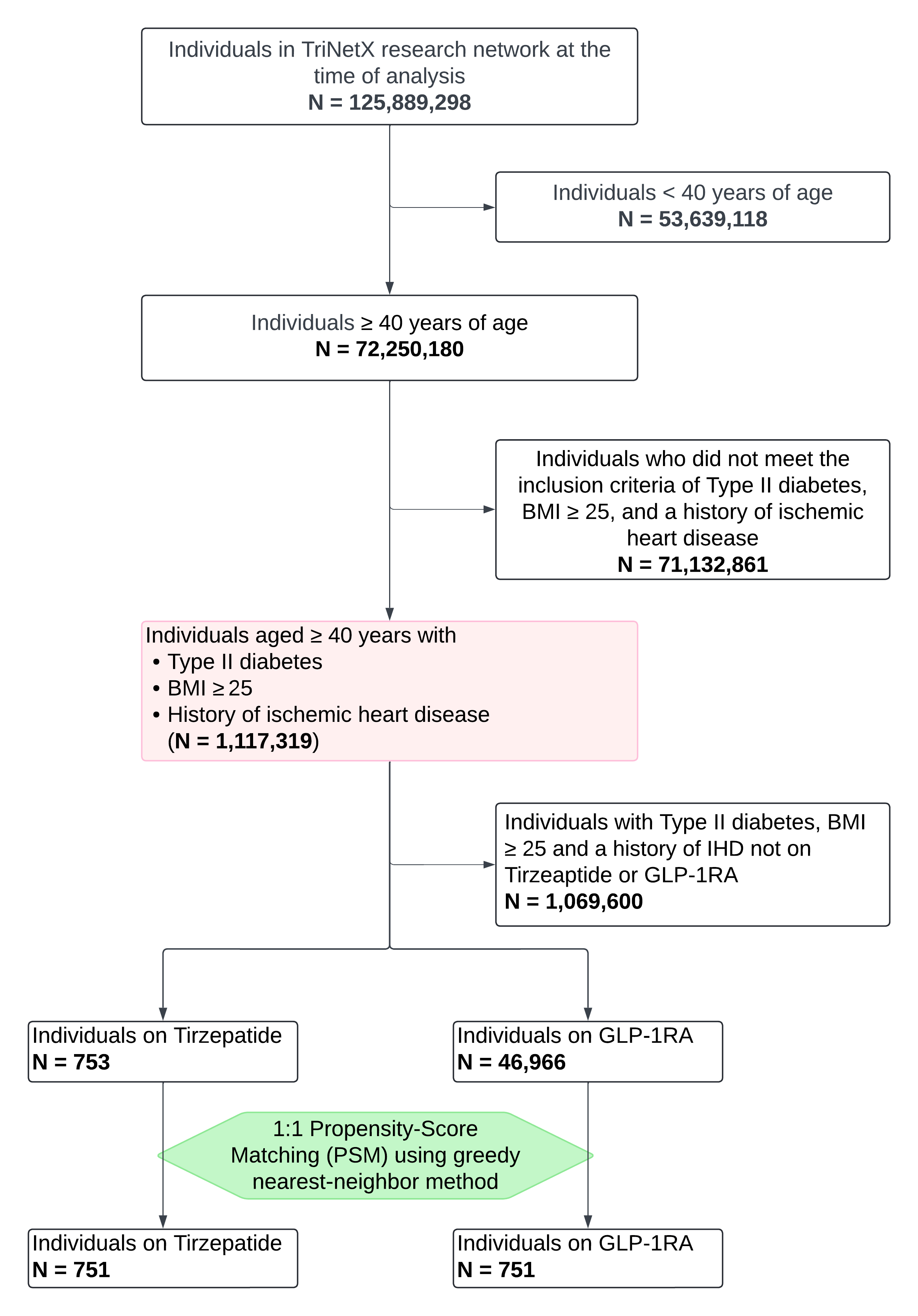

Supplement: Supplementary data [file mmc1.docx]
